# Identification of receptor-binding domains of Bacteroidales antibacterial pore-forming toxins

**DOI:** 10.1016/j.jbc.2025.111113

**Published:** 2025-12-29

**Authors:** Sofia Borgini, Edwin Pasveer, Chloé Petre, Bogdan I. Iorga, Didier Vertommen, Han Remaut, Jean-François Collet, Frédéric Lauber

**Affiliations:** 1 de Duve Institute, Université Catholique de Louvain, Brussels, Belgium; 2 WELBIO Department, WEL Research Institute, Wavre, Belgium; 3 Structural Biology Brussels, Vrije Universiteit Brussel, Brussels, Belgium; 4 Structural and Molecular Microbiology, VIB-VUB Center for Structural Biology, Brussels, Belgium; 5 Université Paris-Saclay, CNRS UPR 2301, Institut de Chimie des Substances Naturelles, Gif-sur-Yvette, France

**Keywords:** Bacteroidales, BSAP, competition, Gram-negative bacteria, gut microbiome, MACPF, outer membrane, pore-forming toxin, protein–protein interaction, receptor

## Abstract

Bacteroidales are abundant Gram-negative bacteria present in the gut microbiota of most animals, including humans, where they carry out vital functions for host health. To thrive in this competitive environment, Bacteroidales use sophisticated weapons to outmatch competitors. Among these, Bacteroidales secreted antimicrobial proteins (BSAPs) represent a novel class of bactericidal pore-forming toxins that are highly specific to their receptor, typically targeting only a single membrane protein or lipopolysaccharide. The molecular determinants conferring this high selectivity remain unknown. In this study, we therefore investigated the model protein BSAP-1 and determined which of its domains is involved in providing receptor specificity. We demonstrate that receptor recognition is entirely driven by the C-terminal domain (CTD) of BSAP-1 using a combination of *in vivo* competition assays, *in vitro* protein binding studies, and mutational analysis. Specifically, we show that deletion of the CTD abrogates BSAP-1 bactericidal activity by preventing receptor binding, whereas grafting the CTD to unrelated carrier proteins enables CTD-driven interaction with the BSAP-1 receptor. Combining structural investigation of a BSAP-1–receptor complex with mutational analysis, we unveil that this interaction is driven by electrostatic interactions. Building upon this discovery, we show that BSAPs can be categorized according to the structure of their CTD, suggesting a strong CTD structure–receptor type correlation. In summary, our research demonstrates that BSAP receptor recognition is driven by their CTD and paves the way for future applications.

Bacteroidales are efficient long-term colonizers of the human intestine and are, on average, the most abundant Gram-negative bacteria in a healthy gut microbiota (1, 2, 3). They play pivotal roles in human health; thanks to their beneficial anti-inflammatory (4, 5), immunomodulatory (6), and metabolic properties (7, 8). To secure their ecological niche in this highly competitive environment, Bacteroidales employ numerous mechanisms to directly antagonize opponents. These include direct injection of toxins *via* the cell contact-dependent type 6 secretion system (T6SS) (9, 10, 11) as well as production of antibacterial molecules, such as Bacteroidales secreted antimicrobial proteins (BSAPs) (12, 13, 14, 15), cholesterol-dependent cytolysin-like (CDCL) proteins (16, 17), bacteroidetocins (18, 19), and the Fab1 (20), BfUbb (21, 22), and BcpT (23) toxins. Among these, the recently identified BSAPs are widespread throughout Bacteroidales and have been shown to actively drive microbiota strain selection, structuring the intestinal flora in space and time (13, 14, 24, 25). Deciphering their mechanism of action at a molecular level, therefore, holds promise to better understand how a healthy human gut microbiota is established and maintained over time.

BSAPs are surface-exposed lipoproteins, *i.e.*, globular proteins anchored to the outside of the cell by an invariable lipidated N-terminal cysteine (26) and targeted to the cell surface by a specific lipoprotein export signal (LES) downstream of that residue (27, 28). In addition, all BSAPs possess a conserved membrane attack complex/perforin (MACPF) domain (Pfam PF01823) (29) and hence belong to the wider MACPF–cholesterol-dependent cytolysin (CDC) superfamily of pore-forming toxins (PFTs) (30). Although highly diverse from a sequence point of view, proteins of this family exhibit strong structural similarity; thanks to the shared MACPF/CDC domain fold. Members include CDCs (31) produced by Gram-positive pathogens, eukaryotic MACPF proteins involved in innate immunity (32, 33), and the recently identified CDCL proteins produced by Bacteroidales (16, 17). Similar to other PFTs, these proteins have the remarkable ability to assemble multimeric membrane-spanning lytic pores from water-soluble, monomeric subunits (34), resulting in the formation of large β-barrel pores of up to 350 Å in diameter (30). Investigation of BSAP-1 (BF638R_1646, GenBank: CBW22174.1), expressed by *Bacteroides fragilis* 638R and targeting a specific outer membrane (OM) β-barrel receptor protein (hereafter B1R^S^) present in a subset of *B. fragilis* strains, revealed that exposure of BSAP-1-sensitive cells to purified BSAP-1 led to a rapid uptake of the cell-impermeable DNA dye propidium iodide (12). Similar to other MACPF/CDC proteins, this strongly suggests that BSAP-1 disrupts the membrane integrity of sensitive cells and that BSAP-1 bactericidal activity results from lytic pore formation. By analogy with characterized members of this group of proteins, the conserved MACPF domain is the likely driving force for BSAP subunit oligomerization and pore formation (30). BSAPs, therefore, represent a novel class of bacterial PFTs.

A striking property of BSAPs is that they are highly specific to their receptor and typically only target a single OM β-barrel protein (12), lipoprotein (15), or lipopolysaccharide (LPS)–lipooligosaccharide (LOS) glycan (13, 14). Due to this high specificity, BSAPs have so far only been described in the context of intraspecies competition, making them the first example of bacterial MACPF proteins possessing bactericidal activity. This contrasts with most other MACPF and CDC proteins that generally display interkingdom activity and use widespread receptor types, such as cholesterol in the case of CDCs (29). The molecular determinants conferring this uniquely high selectivity to BSAPs remain currently unknown.

To address this gap in knowledge, we investigated BSAP receptor recognition and binding. Following maturation and surface translocation, BSAPs are typically composed of three domains: a disordered N-terminal region harboring the lipidated cysteine and LES export sequence; the highly conserved MACPF domain; and one to several C-terminal domains (CTDs) of unknown function (Fig. 1*A*) (35). Because the MACPF domain is essential for BSAP subunit oligomerization and lytic pore formation (30), it is unlikely to additionally be involved in providing specificity to receptors as diverse as proteins or glycans. Instead, sequence conservation analyses showed that CTDs vary greatly, resulting in a wide array of different BSAP architectures (14, 35). As BSAP receptors are likewise highly variable, we therefore hypothesized that high CTD diversity directly correlates with that of BSAP receptors, and that CTDs dictate receptor specificity and are essential for receptor binding.

**Figure 1:**
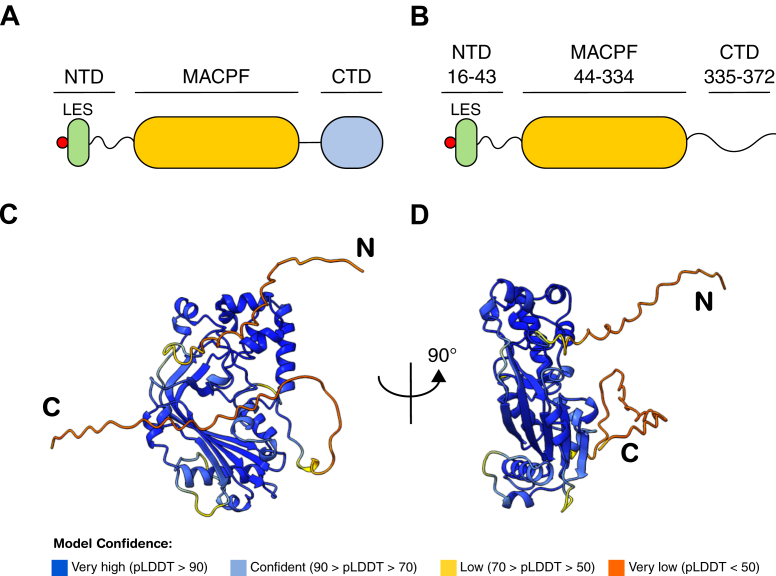
**BSAP proteins are structurally organized into three distinct domains.***A*, domain architecture of a generic BSAP (*A*) and of *Bacteroides fragilis* 638R BSAP-1 (GenBank code: CBW22174.1; *B*). Domain boundaries for BSAP-1 are indicated. The MACPF domain is in *yellow*; the CTD is in *blue*; the LES is in *green*; and the *red* dot denotes the lipidated N-terminal cysteine. *C* and *D*, AlphaFold3 model of BSAP-1. pLDDT is AlphaFold’s per-residue confidence score, which scales from 0 to 100. BSAP, Bacteroidales secreted antimicrobial protein; CTD, C-terminal domain; LES, lipoprotein export signal; MACPF, membrane attack complex/perforin domain; pLDDT, predicted local distance difference test.

Here, we explore this hypothesis and show that CTDs are indeed critical for BSAP receptor recognition. To this end, we used BSAP-1 as our model protein, as it has been shown to actively promote strain exclusion in the gastrointestinal tract by targeting the B1R^S^ receptor crucial for mammalian gut colonization (12, 13). By employing a multidisciplinary approach, we show that the BSAP-1 CTD is both essential and sufficient for receptor binding. Using a structure-based *in silico* analysis pipeline, we further show that BSAPs can be clustered according to the structure of their CTDs, and that each cluster likely targets a distinct type of receptor.

## Results

### The BSAP-1 CTD is essential for *in vivo* bactericidal activity

To determine the importance of CTDs for BSAP functioning, we first tested their relevance for bactericidal activity *in vivo*. To this end, we investigated BSAP-1 produced by *B. fragilis* 638R, which targets the B1R^S^ receptor expressed by other *B. fragilis* strains, including the type strain *B. fragilis* NCTC 9343 (BF9343_1563, GenBank: CAH07344.1). *B. fragilis* 638R itself is protected from BSAP-1 by expressing a homolog of B1R^S^, B1R^R^ (BF638R_1645, GenBank: CBW22173.1), which is not recognized by BSAP-1. AlphaFold3 (36) structural prediction yielded a high-confidence molecular model for B1R^S^ and showed that the protein is likely a member of the FadL family of proteins (PF03349) (37), characterized by a 14-stranded β-barrel plugged by an N-terminal hatch domain and an extracellular domain extending above the membrane plane (Fig. S1). Similar to other members of this family, B1R^S^ might hence be involved in the transport of hydrophobic molecules, such as long-chain fatty acids (38, 39). AlphaFold3 structural modeling of BSAP-1 indicates that the bulk of the mature protein (39.4 kDa) is comprised of the conserved MACPF domain (residues L44–D334), flanked by short regions with low confidence scores at the N-terminal domain (NTD; residues C16–K43) and CTD (residues S335–P372). Intriguingly, the CTD is only 38 amino acids in length and does not appear to adopt a specific fold, raising the question whether this polypeptide is indeed sufficient to confer high receptor specificity to BSAP-1 (Fig. 1, *B*–*D*).

To test this hypothesis, we employed a strategy combining BSAP-1 mutagenesis, followed by *in vivo* phenotyping, as this allows to test bactericidal activity of BSAP-1 variants while also controlling for correct surface localization of the protein. We chromosomally modified *B. fragilis* 638R to express full-length, C-terminally His-tagged, BSAP-1 (BSAP-1-His) from its native locus to monitor expression levels. In parallel, we generated a strain expressing a truncated version missing the CTD (BSAP-1-His ΔCTD). In addition, we engineered a derivative unable to reach the bacterial cell surface by mutating the LES of BSAP-1, substituting the five amino acids immediately downstream of the lipidated cysteine with alanines (residues T17–F21, BSAP-1-His LES∗). Last, to confirm that the NTD of BSAP-1 was not involved in receptor recognition, we generated a derivative in which the N-terminal V23–V36 residues were replaced by a generic α-helical sequence (40) (EEAAAAKEEAAAAK; BSAP-1-His NTD∗). This helix was chosen based on the recent finding that the presence of the NTD is critical for lipoprotein extraction from the inner membrane by the Lol machinery (41); deletion rather than substitution of this region of the protein would hence prevent surface localization.

To assess bactericidal activity, we took advantage of the fact that the BSAP-1 receptor can be expressed in the heterologous host *Bacteroides thetaiotaomicron*, not targeted by BSAP-1 under normal conditions. We then performed competition assays between *B. fragilis* 638R strains expressing BSAP-1 or its derivatives and *B. thetaiotaomicron* competitor strains (*Bt*) expressing the BSAP-1 receptor B1R^S^. Competitor survival was assessed by monitoring the growth of serial dilutions on selective media. Importantly, all variants of *B. fragilis* 638R were constructed in a *tssB–tssC* deletion background (hereafter ΔT6SS), leading to inactivation of the T6SS constitutively expressed by this strain (42), thereby minimizing alternative sources of antagonism and allowing us to attribute the observed phenotypes to BSAP expression and function.

**Figure 2:**
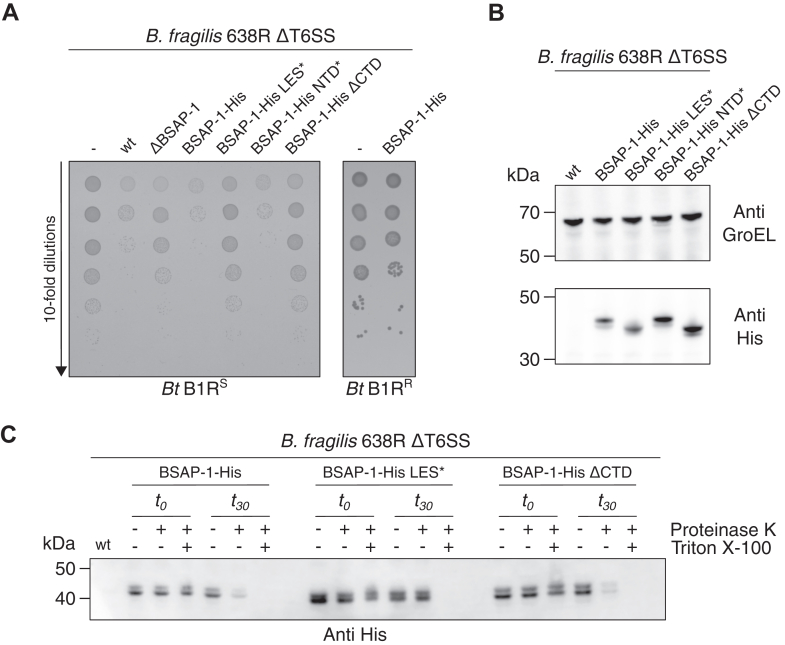
**The BSAP-1 CTD is essential for bactericidal activity *in vivo*.***A*, survival of *Bacteroides thetaiotaomicron* (*Bt*) competitor cells expressing the indicated B1R variant, following incubation with various *Bacteroides fragilis* 638R strains. *B*, whole-cell immunoblot analysis of the indicated *B. fragilis* 638R strains. Detection of GroEL was used as a loading control. *C*, immunoblot analysis of the indicated proteinase K treated *B. fragilis* 638R strains in the absence or the presence of the detergent Triton X-100. Representative results from at least three independent experiments are shown for each panel. BSAP, Bacteroidales secreted antimicrobial protein; CTD, C-terminal domain.

In agreement with previous reports (12, 13), incubation of *B. thetaiotaomicron* expressing B1R^S^ with *B. fragilis* expressing BSAP-1 (wt) or BSAP-1-His led to a ∼3-log reduction in competitor survival compared with the untreated control or to cells incubated with a BSAP-1 deletion strain (ΔBSAP-1) (Fig. 2*A*). Experiments in which *B. thetaiotaomicron* expressed B1R^R^, of which expression was verified by proteomics (Table S1), confirmed that this phenotype is entirely dependent on expression of B1R^S^. As expected, expression of BSAP-1-His LES∗ led to competitor survival similar to that of the untreated control, indicating that surface localization of BSAP-1 is critical for bactericidal activity. On the other hand, the BSAP-1-His NTD∗ construct resulted in competitor killing similar to that of the wt protein, demonstrating that the NTD is not required for receptor recognition. Interestingly, expression of BSAP-1-His ΔCTD resulted in competitor survival comparable to that of the untreated control, indicating that truncation of the BSAP-1 CTD does prevent bactericidal activity (Fig. 2*A*). Whole-cell immunoblotting confirmed that the observed phenotypes are likely because of the mutation directly affecting the functionality of BSAP-1 rather than expression levels or stability of the protein (Fig. 2*B*).

As evidenced by the LES mutant of BSAP-1, surface localization of the protein is a prerequisite for bactericidal activity. To exclude the possibility that truncation of the CTD affects proper localization of the protein, we performed proteinase K surface accessibility assays of cells expressing BSAP-1 and its derivatives. As anticipated, BSAP-1-His was degraded by proteinase K within 30 min of incubation, both in intact cells and cells permeabilized by the detergent Triton X-100 (Fig. 2*C*). On the other hand, BSAP-1-His LES∗ was susceptible to proteinase K only in the presence of detergent, confirming that the protein is indeed trapped within the cells. BSAP-1-His ΔCTD was readily degraded by proteinase K in intact cells, demonstrating that deletion of the CTD does not affect translocation of the protein to the cell surface (Fig. 2*C*). Together, this indicates that the BSAP-1 CTD is not required for correct surface localization of the protein but is critical for bactericidal activity *in vivo*.

### The BSAP-1 CTD is essential for receptor binding

**Figure 3:**
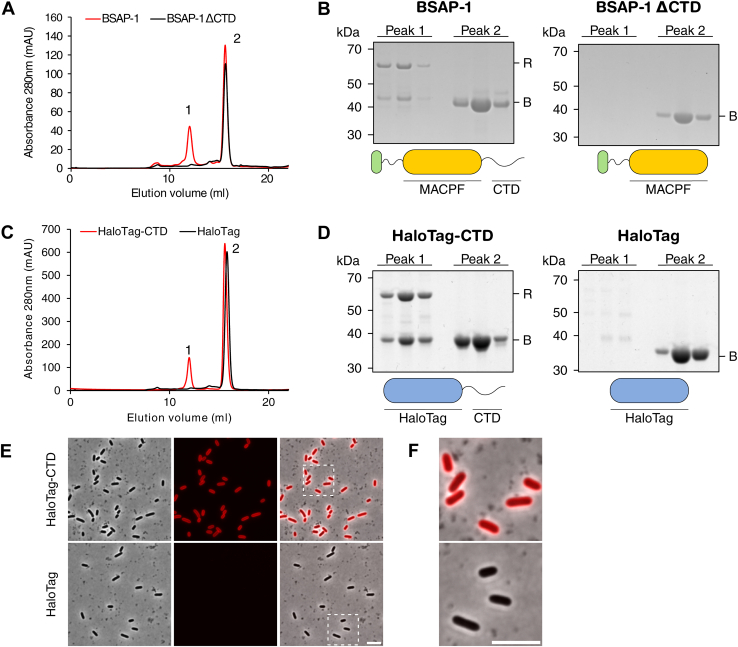
**The BSAP-1 CTD is essential and sufficient for receptor binding.***A*, size-exclusion chromatography profile of affinity-purified BSAP-1 (*red*) or BSAP-1 ΔCTD (*black*) following incubation with a B1R^S^-containing membrane fraction. *B*, Coomassie-stained gel showing the protein content of peak 1 and peak 2 from *A* for BSAP-1 (*left*) and BSAP-1 ΔCTD (*right*), respectively. Schematic representation of each protein is indicated below. MACPF domain is in *yellow*; LES is in *green*. *C*, size-exclusion chromatography profile of affinity-purified HaloTag-CTD (*red*) or HaloTag (*black*) following incubation with a B1R^S^-containing membrane fraction. *D*, Coomassie-stained gel showing the protein content of peak 1 and peak 2 from *C* for HaloTag-CTD (*left*) and HaloTag (*right*), respectively. Schematic representation of each protein is indicated below. HaloTag is in *blue*. *B*, bait protein; R, BSAP-1 receptor. *E*, HaloTag-CTD (*top*) or HaloTag (*bottom*) labeling of *Bacteroides thetaiotaomicron* B1R^S^ cells was visualized by epifluorescence microscopy. The scale bar represents 5 μm. *F*, zoom-in of cells boxed in *E*. The scale bar represents 5 μm. Representative results from at least three independent experiments are shown for each panel. BSAP, Bacteroidales secreted antimicrobial protein; CTD, C-terminal domain; LES, lipoprotein export signal; MACPF, membrane attack complex/perforin domain.

Next, we investigated whether our observed *in vivo* data resulted from the inability of truncated BSAP-1 to bind its receptor or if the formation of lytic pores was prevented. To this end, we recombinantly expressed and purified N-terminally His-tagged BSAP-1 as a soluble protein from *Escherichia coli*, omitting the N-terminal signal peptide and lipidated cysteine (data not shown). As we were unable to generate a functional tagged fusion of the BSAP-1 receptor for affinity purification, we resorted to overexpression of the untagged protein in *B. thetaiotaomicron*, followed by cell lysis and recovery of the total membrane fraction containing the receptor. After confirming bactericidal activity of purified BSAP-1 (Fig. S2), we evaluated BSAP–receptor complex formation by performing a pull-down assay using BSAP-1 and incubating it with the B1R^S^-enriched membrane fraction. Following affinity purification, the elution fraction was injected onto a size-exclusion chromatography column, and peak fractions were analyzed for the presence of BSAP-1 and its receptor. Doing so, we observed two distinct, well-resolved peaks (peak 1 and peak 2) (Fig. 3*A*). SDS-PAGE analysis indicated that peak 2 contained excess free BSAP-1, whereas peak 1 showed that BSAP-1 coeluted with a protein of approximately 60 kDa, in accordance with the theoretical molecular weight of B1R^S^ (62 kDa) (Fig. 3*B*). Proteomics analysis confirmed that this band indeed corresponded to B1R^S^ (Table S2), clearly demonstrating the formation of a stable BSAP-1–receptor complex.

Having successfully validated our experimental approach, we next sought to investigate the effect of CTD truncation on receptor binding. BSAP-1 ΔCTD was purified as above, and, unlike full-length BSAP-1, did not show any bactericidal activity (Fig. S2). The protein was then used as bait for pull-down assays, followed by gel filtration analysis. Only a single peak corresponding to BSAP-1 ΔCTD was observed (Fig. 3, *A* and *B*), showing that the protein was unable to bind its receptor and strongly suggesting that the CTD is essential for BSAP–receptor complex formation.

### The BSAP-1 CTD is sufficient for the formation of a stable BSAP-1–receptor complex

While the aforementioned results supported the idea that the BSAP-1 CTD is directly involved in receptor binding, our results could also be explained by partial misfolding of BSAP-1 following CTD truncation. To rule out this possibility and to clearly demonstrate that the CTD is the sole element required for receptor recognition and binding, we grafted the CTD of BSAP-1 to the unrelated carrier protein HaloTag, resulting in the HaloTag-CTD fusion protein. Similar to full-length BSAP-1, we observed two distinct peaks (peak 1 and peak 2) following pull-down and gel filtration analysis when using purified HaloTag-CTD as bait in our assay (Fig. 3*C*). As previously, peak 2 contained excess free HaloTag-CTD, whereas mass spectrometry (MS) confirmed that the BSAP-1 receptor B1R^S^ and HaloTag-CTD coeluted in peak 1 (Fig. 3*D* and Table S2). Pull-down using a purified HaloTag control protein established that this interaction is specific and dependent on the presence of the BSAP-1 CTD (Fig. 3, *C* and *D*). Identical results were obtained when testing an equivalent fusion to *E. coli* maltose-binding protein (Fig. S3, *A* and *B*), further demonstrating that this interaction is entirely driven by the BSAP-1 CTD. Taking advantage of the fact that HaloTag can be fluorescently labeled, we investigated whether this binding could be recapitulated *in vivo*. To this end, we incubated HaloTag-CTD with exponentially growing *B. thetaiotaomicron* cells expressing B1R^S^, followed by labeling using a HaloTag tetramethylrhodamine dye. Fluorescence microscopy inspection of these cells clearly showed strong surface labeling, which was absent from cells labeled with a HaloTag control protein (Fig. 3, *E* and *F*) or cells expressing B1R^R^ (Fig. S3, *C* and *D*). Identical results were obtained when using *B. fragilis* strain NCTC 9343 endogenously expressing B1R^S^, although with an overall lower fluorescence signal (Fig. S3, *E* and *F*). Taken together, our results demonstrate that the BSAP-1 CTD is the driving force providing receptor specificity and is sufficient for interaction with the BSAP-1 receptor *in vitro* and *in vivo*. In addition, our experiments show that BSAPs can be used to develop novel high-affinity probes to investigate the OM of Bacteroidales.

### BSAP-1 binds to the extracellular domain of B1R^S^

**Figure 4:**
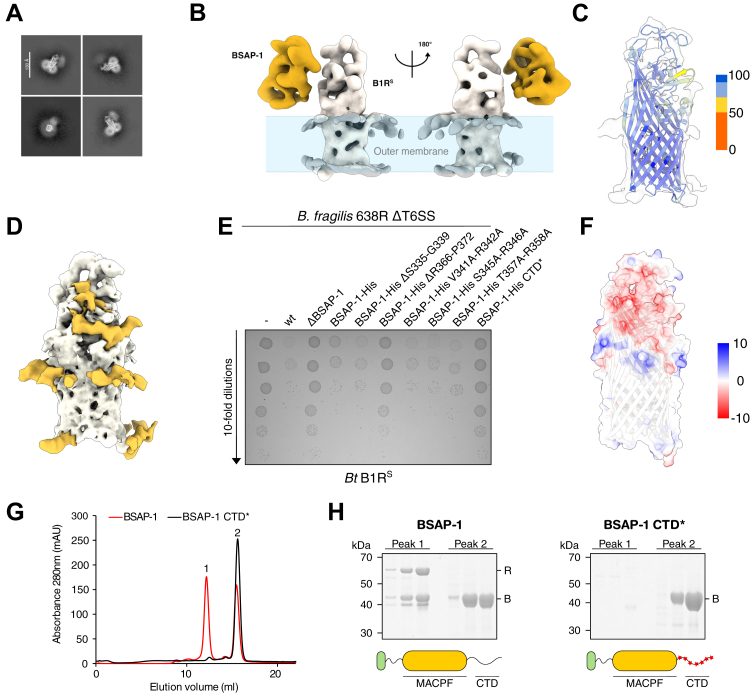
**BSAP-1 and B1R^S^ form a 1:1 complex that is driven by electrostatic interactions.***A*, representative 2D class averages of the BSAP-1–B1R^S^ complex in both side- and top-view orientations. Secondary structural features in B1R^S^ can be distinguished. BSAP-1 occurs as a more diffuse particle. *B*, cryo-EM map of the complex. BSAP-1 is bound to the extracellular part of B1R^S^. *C*, rigid body fitted AlphaFold3 (AF3) model of B1R^S^ inside the density map. AF3 pLDDT confidence score scale is indicated. *D*, density differences between the AF3 model of B1R^S^ and the cryo-EM density map. Density corresponding to the B1R^S^ model is shown in *gray*, and density absent from the AF3 model is indicated in *yellow*. *E*, survival of *Bacteroides thetaiotaomicron* (*Bt*) cells expressing B1R^S^, following incubation with various *Bacteroides fragilis* 638R strains. *F*, surface representation of the B1R^S^ model (semitransparent), colored according to electrostatic potential. Electrostatic potential scale bar is indicated. *G*, size-exclusion chromatography profile of affinity-purified BSAP-1 (*red*) or BSAP-1 CTD∗ (*black*), following incubation with a B1R^S^-containing membrane fraction. *H*, Coomassie-stained gel showing the protein content of peak 1 and peak 2 for BSAP-1 (*left*) and BSAP-1 CTD∗ (*right*), respectively. Schematic representation of each protein is indicated below (*bottom*). MACPF domain is in *yellow*; LES is in *green*; and CTD mutations are indicated by *red star* shapes. BSAP, Bacteroidales secreted antimicrobial protein; CTD, C-terminal domain; LES, lipoprotein export signal; MACPF, membrane attack complex/perforin domain; pLDDT, predicted local distance difference test.

To gain structural insights into the BSAP-1 CTD interaction with the B1R^S^ receptor, peak 1 fractions obtained from a BSAP-1 pull-down were plunge frozen and imaged using single-particle cryo-EM. Particles extracted from the collected micrographs were aligned, and 2D class averages were generated (Fig. 4*A*). The resulting averages display high contrast secondary structural features for B1R^S^, along with a detergent micelle around the transmembrane region of the receptor. However, BSAP-1 appears as a diffuse shape, indicating misalignment because of heterogeneity in its positioning relative to the receptor, reflecting intrinsic conformational flexibility within the complex.

To characterize the organization of the complex, 2D classes showing both BSAP-1 and B1R^S^ were selected for 3D reconstruction. The electron density maps, refined to a resolution of ∼7 Å, revealed the global architecture of the complex in which one BSAP-1 monomer is bound to one receptor molecule (Figs. 4*B*, S4, and Table S3). This indicates that, in our experimental setup, there is no requirement for BSAP-1 oligomerization prior to receptor binding and that pore assembly likely occurs after receptor recognition.

BSAP-1 is positioned on the extracellular side of the receptor, but because of the apparent flexibility and small size of the complex, further refinement to a higher resolution was hindered. However, local 3D refinement focused on B1R^S^ allowed us to obtain a map at 4.45 Å resolution. This allowed rigid body fitting of the molecular model of B1R^S^ predicted by AlphaFold3 (Fig. 4*C*; CC*mask* = 0.59) (43). Interestingly, a difference map subtracting the model from the cryo-EM map revealed unaccounted density corresponding to the detergent micelle as well as a continuous excess density on the extracellular side of the receptor (Fig. 4*D*). The size and elongated shape of the excess density would be compatible with that of an extended polypeptide chain. Although the resolution does not allow *de novo* model building, we therefore hypothesize that this density corresponds to the BSAP-1 CTD. Taken together, our structural analysis reveals that BSAP-1 and its receptor form a 1:1 complex in our *in vitro* conditions and that the globular domain of BSAP-1 shows high conformational flexibility with respect to B1R^S^ in this complex.

### BSAP-1 binding to B1R^S^ is driven by electrostatic interactions

We next examined the CTD to identify residues critical for B1R^S^ binding and/or contributing to the overall BSAP-1 mechanism of action. Doing so, we identified three features potentially involved in this interaction. First, the bulk of the CTD is connected to the conserved MACPF domain through a flexible linker sequence (SSKGG; residues S335–G339) (Fig. S5*A*), potentially involved in the aforementioned observed conformational heterogeneity and possibly important to confer sufficient flexibility to allow oligomer assembly following receptor binding. Second, the C terminus of the CTD is a highly charged heptapeptide, containing three positively charged arginine residues but lacking any negatively charged amino acids (Fig. S5*A*), which might drive electrostatic interactions between the CTD and B1R^S^. Last, we identified three potential proteolytic cleavage sites (V341–R342, S345–R346, and T357–R358) (Fig. S5*A*) of a family of C11-type proteases abundantly produced by Bacteroidales (44, 45, 46), which have been shown to cleave on the C-terminal side of arginine and lysine residues (45, 46, 47). These proteases are lipoproteins anchored to the outer leaflet of the OM and would thus be able to interact with BSAPs. In addition, previous investigation of CDCL proteins from Bacteroidales revealed that proteolytic cleavage by these proteases is essential for their activity (16) and could thus also contribute to BSAP bactericidal activity.

To investigate the contribution of each of these features to B1R^S^ binding and/or BSAP-1 activity, we generated *B. fragilis* strains expressing BSAP-1 deletion mutants for the flexible linker and C-terminal heptapeptide (BSAP-1-His ΔS335-G339 and BSAP-1-His ΔR366-P372, respectively) and substitution variants for the three potential cleavage sites (BSAP-1-His V341A–R342A, S345A–R346A, and T357A–R358A). After confirming correct expression of the proteins (Fig. S5*B*), we performed competition assays against *B. thetaiotaomicron* expressing B1R^S^ (Fig. 4*E*). Neither the flexible linker deletion nor the substitution mutations affected BSAP-1 bactericidal activity, indicating that, in isolation, none of these features is essential for activity. However, deletion of the C-terminal heptapeptide significantly increased competitor survival, although not to the level of the BSAP-1 deletion strain. Immunoblotting confirmed correct expression of the protein (Fig. S5*B*), hence highlighting the importance of this region for receptor binding.

We therefore further investigated the CTD–B1R^S^ interaction interface. Interestingly, the excess density observed in our cryo-EM maps is located in a defined groove on the extracellular domain of B1R^S^, which shows a strong negative electrostatic potential (Fig. 4*F*). Analysis of the full-length CTD sequence shows, similarly to the C-terminal heptapeptide, an enrichment in positively charged residues (6 Arg, 1 Lys out of 38 residues; Fig. S5*A*) in conjunction with a complete absence of negatively charged residues. Similar to the C-terminal heptapeptide, we therefore hypothesized that positively charged residues distributed throughout the BSAP-1 CTD might be required to mediate interaction with the receptor.

To interrogate this model, we generated a *B. fragilis* strain expressing a BSAP-1 derivative, in which all charged residues were replaced by alanine (BSAP-1-His CTD∗). Incubation of this strain with the competitor resulted in a lack of antagonistic phenotype (Fig. 4*E*), although the BSAP-1-His CTD∗ protein was expressed and correctly localized to the cell surface (Fig. S5, *B* and *C*), indicating that mutation of these residues prevents *in vivo* bactericidal activity. Incubation of the competitor strain with recombinantly expressed and purified BSAP-1-His CTD∗ confirmed the lack of bactericidal activity (Fig. S5*D*), and a pull-down assay indicated that this is due to an incapacity to bind to the B1R^S^ receptor (Fig. 4, *G* and *H*).

Taken together, these results demonstrate that BSAP-1 binding is, at least in part, driven by electrostatic interactions between its CTD and B1R^S^.

### The CTD is the major structural determinant for the classification of BSAPs

**Figure 5:**
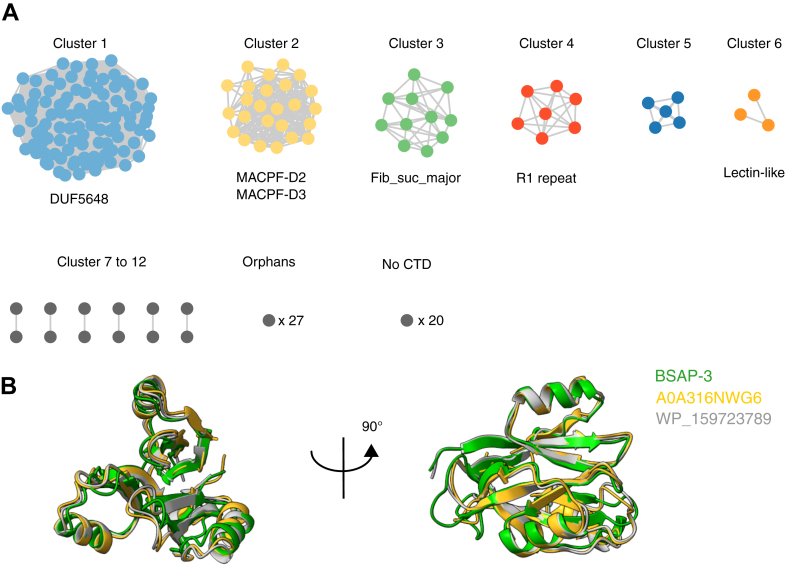
**BSAPs can be clustered according to the structure of their CTD.***A*, network analysis of BSAPs clustered according to structural similarity of their CTDs. Where possible, Foldseek-derived CTD annotation is indicated below each cluster. *B*, front (*left*) and side (*right*) views of the structural comparison between AlphaFold3 models of the CTD of BSAP-3 (*green*), the PF18885 representative A0A316NWG6 (*gold*), and the DUF5648 domain of WP_159723789, a previously described GH70 α-glucanotransferase from *Enterococcus* sp. CSURQ0835 (*gray*). BSAP, Bacteroidales secreted antimicrobial protein; CTD, C-terminal domain; GH70, glycoside hydrolase family 70.

BSAPs are able to recognize vastly different receptor types, such as OM proteins or LPS–LOS glycans, with which they must engage in highly specific interactions in order to eliminate their targets with high selectivity. Having demonstrated that the CTD of BSAP-1 is required and sufficient for this purpose, we wanted to confirm that this is a common feature and that the CTD drives receptor specificity across diverse BSAPs. Specifically, we aimed at determining if BSAPs could be classified according to their CTD and if this could inform on the type of receptor that they target. While a previous work used full-length sequences and amino acid identity scores to classify BSAPs (14), we reasoned that a structure-based comparison focusing solely on CTDs, less sensitive to sequence variation, might identify shared features among BSAPs targeting similar receptors. To this end, we recovered the full-length sequences of all proteins with an MACPF domain (PF01823) belonging to the Bacteroidota phylum from the InterPro database (48). Following multiple sequence alignment and clustering, a representative sequence for each cluster was trimmed to encompass only the CTD, and its structure was predicted using AlphaFold3. Last, we performed an all-against-all structural comparison using Foldseek (49), allowing us to classify CTDs according to their structural similarity (Table S4). This resulted in the generation of a network of 214 CTDs split into 12 distinct groups (Fig. 5*A* and Table S4), with the largest one, group 1, comprised of 103 representative sequences and accounting for 48.6% of all clusters.

Manual inspection revealed that all group 1 CTDs are characterized by a domain of unknown function (DUF5648, Pfam PF18885) widespread throughout the bacterial kingdom and adopting a three-bladed β-α propeller architecture (Fig. 5*B*). Of note, this group contained homologs to the CTDs of BSAP-2, BSAP-3, and M068_0191, three previously described BSAPs targeting the O-antigen glycan chain of their respective target strains (13, 14), suggesting that other members of this group could likewise target O-antigen glycans. In support of this hypothesis, the DUF5648 domain has recently been identified in α-glucanotransferase enzymes belonging to the wider glycoside hydrolase family 70 (Fig. 5*B*) (50), further suggesting that this domain could be involved in glycan recognition and binding. This strongly indicates that BSAPs targeting the same type of receptor share an overall similar structure.

Among the remaining groups described in Fig. S6 and Table S4, group 2 (25 sequences) (Fig. 5*A*) was comprised of CTDs structurally identical to that of Bt_3439 (Protein Data Bank code: 3KK7) (35), the currently only available structure of a BSAP. This CTD is composed of two individual domains of unknown function, D2 and D3 (Pfam PF20779 and PF20785, respectively), which are taxonomically restricted to Bacteroidota and lack structural homologs (Fig. S5*A*). The receptor of Bt_3439 remains currently unknown.

Besides these 12 groups, we also identified 27 “orphan” BSAPs, of which the CTDs have no structural homologs within our dataset and were hence not further investigated (Table S4). Of note, these included BSAP-1 and BSAP-4, both shown to target OM proteins (12, 15). In the case of BSAP-1, the lack of identified structural homologs is likely because of the short, unstructured nature of its CTD, rendering structural comparison challenging. In the case of BSAP-4, this could suggest that Bacteroidota have evolved novel protein-binding domains in order to specifically antagonize competitors.

Our structural analysis of BSAP CTDs allowed clustering of more than 90% of representative sequences in our dataset (194/214 sequences). Curiously, however, manual inspection of the remaining 20 proteins (9% of total representatives) showed that these BSAPs possess no CTD at all, with the protein sequence stopping shortly after the end of the conserved MACPF domain. To clarify how these BSAPs recognize their receptor, we extracted and structurally predicted the NTD of the above analyzed 214 representative proteins. Similarity clustering revealed that more than 80% of BSAPs (177/214 sequences) do not possess a structured NTD other than the disordered tether region harboring the LES signal crucial for surface localization. The remaining sequences fell into two distinct groups, with group 1 NTDs representing 16% of total representatives (35 sequences), whereas group 2 NTDs only represented 1% (2 sequences) (Fig. S7*A* and Table S4).

Group 1 NTDs adopt an eight-stranded, immunoglobulin-like β-sandwich fold known as the Bacteroides-associated carbohydrate-binding often N-terminal (BACON) domain (Pfam PF13004) (Fig. S7*B*) (51). While initially described as a carbohydrate-binding module because of it being found in combination with domains catalytically active on carbohydrates, a more recent study found no experimental evidence for substrate binding or in mediating protein–protein interactions for the BACON domain of the endoxyloglucanase BoGH5A (52). Instead, the authors suggest that the BACON domain might provide the enzyme with additional mobility by distancing the catalytic module from the cell surface. As a result, whether the BACON domain directly participates in receptor recognition and, more generally, how it contributes to BSAP-mediated killing, remains currently unknown.

Crossexamination of our CTD and NTD clustering results revealed two distinct trends. First, none of the group 1, 2, 4, or 6 CTD-containing BSAPs are endowed with a structured NTD (Fig. S7*D*), strongly suggesting that substrate recognition is entirely driven by their CTD. On the other hand, all group 3, 7, and 11 CTD-containing BSAPs possess a group 1 NTD, indicating an essential function of this domain for their functioning. Whether this includes interaction with their receptor remains to be determined. Cluster 1 NTDs are further found sporadically throughout the remaining BSAPs, in particular in 12 representatives (5.6% of sequences) lacking a CTD. Taken together, our analysis showed that BSAPs can be categorized into distinct groups based on the 3-dimensional structure of their CTD, and that BSAPs within the same group likely target the same type of receptor. In addition, several CTD structural groups consistently co-occur with group 1 NTD, suggesting a potential coevolution between these domains. Last, only eight representative sequences in our dataset (3.7% of total sequences) possess neither a distinct NTD nor a distinct CTD (Fig. S7*D*); how these proteins recognize and bind their receptors needs to be determined.

## Discussion

BSAPs are a novel class of PFTs widespread throughout the phylum Bacteroidota, which are crucial in mediating bacterial antagonism, including in the human gut (14, 25). BSAPs are highly specific to their receptor, typically targeting only a single OM protein or LPS–LOS glycan variant. This makes them potent weapons for intraspecies competition, enabling the producing strain to outcompete its opponents and secure its ecological niche. However, while several BSAP–receptor pairs have been identified, current knowledge falls short in providing insights into how a given BSAP recognizes and binds its receptor with high selectivity.

In this work, we investigated the model protein BSAP-1 and defined which of its domains is involved in providing receptor specificity. We clearly demonstrate that BSAP-1 receptor recognition is entirely driven by the 38-amino acid–long CTD of BSAP-1 using a combination of *in vivo* competition assays, *in vitro* protein binding studies, and fluorescence microscopy. Specifically, we show that deletion of the CTD abrogates BSAP-1 bactericidal activity by preventing receptor binding, whereas grafting the CTD to unrelated carrier proteins, such as HaloTag and maltose-binding protein, enables CTD-driven interaction with the BSAP-1 receptor.

Although not at atomic resolution, volumetric analysis of our cryo-EM density map nonetheless allows to locate the CTD-binding site within a negatively charged groove on the extracellular domain of B1R^S^. In conjunction with mutagenesis analyses demonstrating that positively charged residues distributed throughout the CTD are crucial for interaction with B1R^S^, we conclude that the formation of this complex is at least partly dependent on electrostatic interactions. Apart from this short density in contact with the B1R^S^ receptor, the bulk of BSAP-1 is seen as a diffuse density on the side of the receptor, indicating substantial conformational flexibility in the BSAP-1–B1R^S^ contact. In addition, our dataset did not provide evidence of BSAP-1-derived density within or in contact with the B1R^S^-containing detergent micelle. Together with the apparent flexibility of BSAP-1 in our complex, this suggests that in our experimental conditions, receptor binding alone is not sufficient to trigger membrane insertion of BSAP-1 and that it does not oligomerize before encountering its receptor. This could be the result of our experimental setup not being conducive for pore assembly, for example, using a detergent-solubilized B1R^S^ fraction rather than membranous vesicles or intact cells. The specific charge and chemistry of the OM bilayer may be needed to trigger oligomerization and conformational transition in BSAP-1. Alternatively, an external trigger might be required to induce BSAP-1 oligomerization, such as the proteolytic activation required for CDCL bactericidal activity (16). It therefore remains unclear if a single receptor binding event is sufficient to trigger BSAP-1 oligomerization, or if each BSAP-1 subunit requires receptor binding prior to pore assembly.

Building upon our discovery, we employ a structural modeling approach to investigate a large collection of BSAP sequences. Our analysis shows that the majority of BSAPs can be categorized according to the predicted structure of their CTD, resulting in 12 distinct groups, and that BSAPs within the same group are likely to target the same type of receptor. To confirm and take advantage of these findings, future investigations should focus on the identification of the receptor type for each BSAP CTD group, which can be inferred from analysis of the genomic neighborhood of the BSAP-encoding gene (13). In addition, this study lays the groundwork for a more in-depth analysis of BSAPs and how receptor selectivity operates at a molecular level. Expanding the repertoire of high-resolution BSAP–receptor complexes will be an invaluable tool to clearly dissect their interactions in the future.

Our analysis shows that many BSAPs (CTD groups 1, 6, 7, 9, 10, and 12; 53% of total representative sequences) are predicted to target glycans in order to carry out their bactericidal function. Interestingly, Bacteroidota have been shown to O-glycosylate many of their proteins, including cell surface–exposed proteins, such as lipoproteins (53, 54). In addition, LPS–LOS glycan biosynthesis regions are typically diverse within a given Bacteroidales species, resulting in a variety of different serotypes (14). Last, it is well documented that Bacteroidales are endowed with numerous phase–variable polysaccharide capsule biosynthesis operons, which show high intraspecies diversity (55, 56). For example, comparison of three *B. fragilis* genomes led to the identification of 28 distinct capsule biosynthesis operons (57). While currently only the O-antigen glycan has been shown to serve as a receptor for BSAPs, this high variability in cell surface structures could hence indicate that glycans, including capsules and protein O-glycosylation moieties, might represent a preferred target for BSAPs.

Our analysis also revealed that a small proportion of BSAPs is endowed with a characteristic N-terminal BACON domain upstream of the conserved MACPF domain. While this domain is widespread throughout the Bacteroidota phylum, its precise function is still unknown. As a result, whether and how the BACON domain participates in receptor binding, and more generally in the overall BSAP mechanism of action, requires further investigation.

Our data show that the CTD of BSAP-1 can be repurposed to generate tools for the investigation of Bacteroidota biology. Expanding this analysis to other BSAPs, targeting diverse receptors such as the LPS–LOS glycan, will provide the opportunity to develop a novel toolbox to study membrane biogenesis in Bacteroidota, a field currently still in its infancy. Indeed, although recent investigations have started to shed light on how members of this phylum build their cell envelope (58, 59, 60, 61), stark differences regarding the machineries used in OM biogenesis as well as its composition exist between Bacteroidota and the model α-proteobacterium *E. coli*. Notably, this includes the production of eukaryotic-like sphingolipids (62), a high proportion of surface-exposed lipoproteins (27, 63), and a unique β-barrel assembly machinery (60, 61). Akin to previously described methodologies relying on modified colicins (64), BSAP or BSAP CTD derivatives could hence be used to investigate these aspects of Bacteroidota cell biology, underscoring their potential as research tools.

Our results demonstrate that the majority of BSAPs is broadly organized into two distinct domains, with the MACPF domain being responsible for bactericidal activity, whereas the CTD provides receptor specificity. BSAPs therefore exhibit an interesting duality, combining a generic mode of action with high selectivity. Future work focusing on CTD engineering could therefore aim to widen and/or modify the tropism of a given BSAP, enabling it to target and kill various pathogenic species. In light of the global rise of antimicrobial resistance and the resulting threat to human health, having access to molecules specifically designed to target pathogens with high specificity, rather than using broad-range antibiotics, represents an invaluable asset. As BSAPs are able to target both proteins and glycans, two main components of the bacterial OM, BSAPs hence possess a strong potential for the development of novel antimicrobial molecules and warrant further investigation.

## Experimental procedures

### Bacterial strains and growth conditions

All strains and plasmids used in this work are listed in Table S5. *Bacteroides* strains were routinely grown anaerobically in brain heart infusion (BHI; BD) supplemented with 1 g/l cysteine, 5 mg/l hemin, and 1 mg/l menadione (BHIs) or modified Gifu anaerobic medium (mGAM; Shimadzu Diagnostics Europe) at 37 °C without shaking. *E. coli* strains were routinely grown aerobically in lysogeny broth (LB; MP Biomedicals) at 37 °C with agitation at 200 rpm. Where required, antibiotics were added at the following concentrations: 200 μg/ml ampicillin for *E. coli* and 5 μg/ml erythromycin and 200 μg/ml gentamicin for *Bacteroides* strains.

### Genetic constructs

Plasmids were constructed by standard Gibson cloning or Q5 site–directed mutagenesis (New England Biolabs) using the primers and target DNA listed in Table S5. For heterologous expression of BSAPs in *E. coli*, expression vectors were constructed by cloning the gene of interest, excluding the signal peptide and conserved cysteine, into a pET15b-TEV derivative in frame with an N-terminal 6-His tag and a Ser-Ser-Gly linker. The MBP and HaloTag coding sequences were amplified from pMAL-c6T and *Flavobacterium johnsoniae* FL_147 genomic DNA, respectively. *Bacteroides* suicide plasmids to introduce in-frame unmarked deletions were produced by cloning the 500 to 1000 bp upstream and downstream flanking regions of the genes of interest into pSIE1 (a gift from Andrew Goodman, Addgene plasmid #136355; http://n2t.net/addgene:136355; Research Resource Identifier: Addgene_136355) (65). *Bacteroides* suicide plasmids to introduce chromosomal modifications were constructed by cloning the gene of interest and the 500 to 1000 bp upstream and downstream flanking regions into pSIE1, followed by site-directed mutagenesis. For the generation of BSAP-1 CTD∗, a gBlocks fragment (IDT) spanning the C-terminal 114 bp of the BSAP-1 gene in frame with a 6-His tag and harboring the desired mutations was ordered and inserted into pFL281 by standard Gibson cloning. *Bacteroides*-integrative expression plasmids were produced by cloning the gene of interest into pWW3452 in place of superfolder GFP (66). All plasmid constructs were confirmed by sequencing.

Suicide and expression plasmids were introduced into the appropriate *Bacteroides* background by biparental mating using *E. coli* S17-1 λpir as donor strain as previously described (67). Briefly, 20 ml of mGAM were inoculated at a 1:100 dilution with an overnight culture of *Bacteroides* and grown anaerobically for 3 h. In parallel, 3 ml LB were inoculated at a 1:100 dilution with an overnight culture of *E. coli* and grown aerobically for 3 h. Cells were collected by centrifugation and washed once with PBS. The pellets were then combined, spotted onto BHI agar, and incubated aerobically overnight at 37 °C. The next day, the cells were recovered, resuspended in PBS, and serial dilutions were plated onto erythromycin and gentamycin containing BHI agar to select for chromosomal plasmid integration. For in-frame deletion and chromosomal tagging suicide vectors, one of the resulting clones was grown overnight in mGAM without antibiotics to allow for loss of the plasmid backbone, diluted 1:100 into fresh medium, grown for 3 to 4 h, and then plated on BHI agar containing 100 μg/l anhydrotetracycline. Anhydrotetracycline-resistant colonies were screened by PCR for the presence of the desired chromosomal modification. All mutant strains were confirmed by sequencing.

### Expression and purification of recombinant proteins

BSAP-1 and its derivatives were purified from *E. coli* BL21 (DE3) cells. The cells were grown in 3.2 l LB medium at 37 °C to an absorbance of 0.5 at 600 nm, and protein expression was induced by the addition of 400 μM IPTG. The cells were then cultured for an additional 3 h at 37 °C. Cells were harvested by centrifugation at 7500*g* for 30 min and stored at −20 °C until further use. All purification steps were carried out at 4 °C. Cell pellets were resuspended in buffer A (50 mM HEPES, 150 mM NaCl, 20 mM imidazole, 1 mM DTT, pH 7.5) containing 30 μg/ml DNase I, 400 μg/ml lysozyme, and cOmplete Protease Inhibitor Cocktail (Merck Life Science) at a ratio of 5 ml of buffer to 1 g of cell pellet. Cells were incubated on ice for 30 min with constant stirring before being lysed by three passages through a French pressure cell at 24,000 PSI. Cell debris was removed by centrifugation at 40,000*g* for 30 min. The supernatant was then diluted to 125 ml with buffer A, clarified using a Stericup Quick Release Millipore Express PLUS 0.22 μm PES filter device (Millipore), and circulated through a 5 ml HisTrap HP column (Cytiva) for 2 h. The column was washed with 12 column volumes (CVs) of buffer A, and bound proteins were eluted with a 20 to 500 mM linear gradient of imidazole over 20 CVs of buffer A. Peak fractions were collected and concentrated to 2.5 ml using a 10-kDa molecular weight cutoff (MWCO) Vivaspin 6 centrifugal filter unit (Sartorius), then injected onto a HiLoad 16/60 Superdex 75 PG column (Cytiva) previously equilibrated in buffer B (50 mM HEPES, 150 mM NaCl, 1 mM DTT, pH 7.5). Peak fractions were concentrated using a 10-kDa MWCO Vivaspin 6 centrifugal filter unit, and protein concentration was determined using a Microplate BCA Protein Assay Kit (Thermo Scientific) according to the manufacturer’s instructions. Glycerol was added to a final concentration of 10%, and the proteins were aliquoted into 50 μl fractions before being snap-frozen and stored at −80 °C until further use.

### Isolation of whole membrane fractions

The total membrane fraction of strain sFL153 overexpressing the BSAP-1 receptor was isolated as follows. A preculture of sFL153 was grown in mGAM-containing erythromycin for 8 h at 37 °C before being used to inoculate 2.4 l of the same medium at a 1:1000 dilution. The cells were then cultured for an additional 16 h at 37 °C. Cells were harvested by centrifugation at 7500*g* for 30 min and stored at −20 °C until further use. All subsequent steps were carried out at 4 °C. Cell pellets were resuspended in buffer B (50 mM HEPES, 150 mM NaCl, 1 mM DTT, pH 7.5) containing 30 μg/ml DNase I and cOmplete Protease Inhibitor Cocktail (Merck Life Science) at a ratio of 5 ml of buffer to 1 g of cell pellet. Cells were incubated on ice for 30 min with constant stirring before being lysed by three passages through a French pressure cell at 24,000 PSI. Cell debris was removed by centrifugation at 40,000*g* for 30 min. The supernatant was recovered, and total membranes were collected by centrifugation at 180,000*g* for 60 min. Membranes were resuspended in buffer B, and protein concentration was determined using a Microplate BCA Protein Assay Kit according to the manufacturer’s instructions before being stored at −20 °C until further use.

### BSAP-1 pull-down assay

For BSAP-1 (and derivatives thereof) and BSAP-1 receptor interaction studies, 2 mg of purified protein was mixed with 60 mg of total protein of B1R^S^-containing membranes in buffer A in a final volume of 10 ml. The mixture was incubated for 30 min at room temperature with constant agitation. Membranes were then solubilized by addition of *n*-dodecyl-β-d-maltopyranoside (DDM; Anatrace) to a final concentration of 1% (w/v) and incubation for 2 h at 4 °C. Insoluble material was removed by centrifugation at 40,000*g* for 30 min. The solution was then circulated three times through 500 μl of HisPur Nickel–Nitrilotriacetic Acid Resin (Thermo Scientific). The column was washed with 10 CVs of buffer A containing 0.03% DDM, and bound proteins were eluted with 8 CVs of buffer A containing 500 mM imidazole and 0.03% DDM. The eluate was concentrated to 500 μl using a 10-kDa MWCO Vivaspin 6 centrifugal filter unit and then injected onto a Superdex 200 Increase 10/300 GL column (Cytiva) previously equilibrated in buffer B containing 0.03% DDM. Peak fractions were collected and analyzed by Coomassie staining.

### Competition assays

For competition assays between *B. fragilis* and *B. thetaiotaomicron* competitor strains, 5 ml mGAM were inoculated at a 1:100 dilution with overnight cultures of *Bacteroides* and grown anaerobically for 5 h. One milliliter of each culture was collected by centrifugation, cells were washed once in PBS, centrifuged again, and normalized according to their absorbance at 600 nm using PBS. *B. thetaiotaomicron* competitor and *B. fragilis* strains were then mixed at a 1:9 ratio, 5 μl spotted onto BHIS agar, and incubated overnight. The next day, the cells were recovered, resuspended in PBS, and serial dilutions were spotted onto erythromycin-containing BHI plates to select for surviving *B. thetaiotaomicron* competitor cells.

### *In vitro* susceptibility assay

Bactericidal activity of purified proteins was assessed as follows. Five milliliters of mGAM were inoculated at a 1:100 dilution with an overnight culture of *Bacteroides* and grown anaerobically for 5 h. A total volume of 350 μl of culture was then mixed with 3.5 ml of Top Agar (BHIs supplemented with 7% agar) and poured over a BHI plate. Sensi-Disc (BD) impregnated with 15 μg of protein of interest was then deposited on top of the agar, before overnight incubation at 37 °C. The next day, plates were imaged using an ImageQuant LAS 500 Camera (GE Healthcare Life Sciences).

### Proteinase K surface accessibility assay

Surface exposure of BSAP-1 and its derivatives was assessed using a protease accessibility assay. Briefly, 20 ml of mGAM were inoculated at a 1:100 dilution with an overnight culture of *Bacteroides* and grown anaerobically for 5 to 6 h. Quantities equivalent to 8 ml of an absorbance of 1 at 600 nm were collected by centrifugation before being resuspended in 640 μl of PBS containing 10 mM MgCl_2_ and 1 mM CaCl_2_.

Cell suspension aliquots of 80 μl were supplemented as appropriate with 200 μg/ml proteinase K (Merck Life Science) and 1% v/v Triton X-100 (Merck Life Science) and incubated for 30 min at room temperature. Reactions were stopped by the addition of 5 mM PMSF (Merck Life Science) and incubation for 5 min, followed by the addition of SDS-PAGE sample buffer containing 1 mM DTT and further incubation at 96 °C for 10 min before analysis by immunoblotting.

### Immunoblotting

The following commercial antisera were used: anti-HisTag peroxidase conjugate (1:4000 dilution, A190-114P; Bethyl) and anti-rabbit IgG peroxidase conjugate (1:5000 dilution, NA-934; Cytiva). GroEL antibodies (1:10,000 dilution) were raised in rabbits against the purified recombinant GroEL protein (68).

For whole-cell immunoblots, *Bacteroides* strains were cultured in mGAM to an absorbance of 0.5 to 0.6 at 600 nm, normalized by absorbance at 600 nm, pelleted by centrifugation, and resuspended in SDS-PAGE sample buffer. Samples were then heat-denatured for 10 min at 96 °C before being separated on precast polyacrylamide NuPAGE Bis–Tris 10% gels (Life Technologies) using NuPAGE MES SDS Running Buffer (Life Technologies). Proteins were transferred onto nitrocellulose membranes using iBlot Transfer Stacks in an iBlot 2 Gel Transfer Device (Life Technologies). The membranes were then blocked with a 2% milk powder solution in Tris-buffered saline containing 0.1% Tween-20 (v/v) for 2 h before being incubated with primary and secondary antibodies as required. Proteins of interest were detected using Immobilon Western HRP Substrate (Merck Life Science) and an ImageQuant LAS 500 Camera (GE Healthcare Life Sciences).

### Epifluorescence microscopy

Fluorescence labeling of *Bacteroides* strains was carried out as follows. Five milliliters of mGAM were inoculated at a 1:100 dilution with an overnight culture of *Bacteroides* and grown anaerobically for 5 h. One milliliter of the culture was recovered, and cells were collected by centrifugation and resuspended in 250 μl mGAM. Twelve micrograms of HaloTag-CTD or HaloTag control protein were added, and the cells were incubated anaerobically at 37 °C for 30 min. Cells were then washed three times with PBS before the addition of 1 μl of a 50 μM HaloTag tetramethylrhodamine ligand (Promega) stock solution and further anaerobic incubation for 20 min in the dark. Cells were washed two times in PBS, followed by three washes in mGAM. Finally, 1 μl of cells was spotted onto mGAM low-melt agarose pads previously prepared using 65 μl Gene Frames (Thermo Scientific).

Phase contrast and fluorescence microscopy images were acquired on a Ti2-E fully motorized inverted epifluorescence microscope (Nikon) equipped with a CFI Plan Apochromat λ DM 100 × 1.45/0.13 mm Ph3 oil objective (Nikon), a Sola SEII FISH Illuminator (Lumencor), a Prime BSI camera (Photometrics), a temperature-controlled and light-protected enclosure (Okolab), and a filter cube for mCherry (32 mm, excitation 562/40, dichroic 593, emission 640/75; Nikon). Image acquisition was controlled by the NIS-Ar software (Nikon). All fluorescence images were acquired at 37 °C with 30% power output and 100 ms exposure time.

Images were processed with Fiji (distribution of ImageJ, version 1.54e) (69), using identical settings for fluorescence contrast and brightness across all regions of interest within the same figure panel, unless indicated otherwise.

### Mass spectrometry

For analysis of proteins following size-exclusion chromatography, samples were heat-denatured for 10 min at 96 °C before being separated on precast polyacrylamide NuPAGE Bis–Tris 10% gels using NuPAGE MES SDS Running Buffer. Protein bands were visualized by Coomassie Blue staining and in-gel digested with trypsin (Promega). To confirm expression of B1R^S^ and B1R^R^ in *B. thetaiotaomicron*, total membrane fractions were prepared as described above, except that a total culture volume of 400 ml was used. Protein concentration was determined using a Microplate BCA Protein Assay Kit (Thermo Fisher Scientific) according to the manufacturer’s instructions, and 15 μg of total protein were separated on a precast polyacrylamide NuPAGE Bis–Tris 10% gel. Protein bands were visualized by Coomassie Blue staining, and a gel slice corresponding to the region of 60 kDa was excised and in-gel digested with trypsin (Promega). Peptides were extracted with 0.1% trifluoroacetic acid in 65% acetonitrile and dried in a SpeedVac.

Peptides were dissolved in solvent A (0.1% trifluoroacetic acid in 2% acetonitrile), directly loaded onto a reversed-phase precolumn (Acclaim PepMap 100; Thermo Scientific) and eluted in backflush mode. Peptide separation was performed using a reversed-phase analytical column (Acclaim PepMap RSLC, 0.075 × 250 mm; Thermo Scientific) with a linear gradient of 4% to 27.5% solvent B (0.1% formic acid in 80% acetonitrile) for 40 min, 27.5% to 50% solvent B for 20 min, 50% to 95% solvent B for 10 min, and holding at 95% for the last 10 min at a constant flow rate of 300 nl/min on an Ultimate 3000 RSLC system. The peptides were analyzed by an Orbitrap Exploris240 mass spectrometer (Thermo Fisher Scientific). The peptides were subjected to NSI source followed by tandem mass spectrometry (MS/MS) in Exploris240 coupled online to the nano-LC. Intact peptides were detected in the Orbitrap at a resolution of 60,000. Peptides were selected for MS/MS using higher energy collisional dissociation setting at 30, ion fragments were detected in the Orbitrap at a resolution of 15,000. A data-dependent procedure that alternated between 1 MS scan followed by MS/MS scans was applied for 3 s for ions above a threshold ion count of 1.0 × 10^4^ in the MS survey scan with 30.0 s dynamic exclusion. MS1 spectra were obtained with an automatic gain control target of 4 × 10^5^ ions and a maximum injection time set to auto, and MS2 spectra were acquired with an automatic gain control target of 5 × 10^4^ ions and a maximum injection time set to auto. For MS scans, the *m/z* scan range was 350 to 1800. The resulting MS/MS data were processed using Sequest HT search engine within Proteome Discoverer 2.5 SP1 against a *B. fragilis* NCTC 9343 or *B. thetaiotaomicron* VPI-5482 protein database obtained from UniProt. Trypsin was specified as a cleavage enzyme allowing up to two missed cleavages, four modifications per peptide and up to five charges. Mass error was set to 10 ppm for precursor ions and 0.02 Da for fragment ions. Oxidation on Met (+15.995 Da), conversion of Gln (−17.027 Da) or Glu (−18.011 Da) to pyro-Glu at the peptide N term, and acrylamide modification of Cys (+71.037 Da) were considered as variable modifications. False discovery rate was assessed using Percolator, and thresholds for protein, peptide, and modification site were specified at 1%.

### Bioinformatic and structural analysis

All sequences possessing a “MAC/Perforin domain” (Pfam PF01823) belonging to the Bacteroidota class were retrieved from the InterPro database (accessed on November 27, 2023), resulting in a total of 617 sequences. After a filtering step to keep only the 606 sequences with complete N termini (starting with a Met), the sequences were aligned using Clustal Omega, version 1.2.4 (70). The MACPF domain was removed, and the remaining NTDs and CTDs were clustered separately using CD-HIT, version 4.8.1 (71) at 95% sequence identity, leading to 280 and 270 clusters, respectively. For the representative sequence of each cluster, a 3D model was generated using the ColabFold (72) implementation of AlphaFold3 (36). Using these structures, a similarity matrix was built based on the fold similarity computed with Foldseek (49), followed by clustering with Cytoscape, version 3.10.1 (https://cytoscape.org/index.html) (73), using a qtmscore of 0.65 as the cutoff value. The full dataset is available through the GitHub account of the Iorga lab (https://github.com/IorgaLab/BSAP-Dataset).

To identify possible BSAP targets, the UniProt annotation of one representative sequence for each NTD or CTD cluster was crossreferenced with Foldseek searches for structural homologs. Where possible, Protein Data Bank–derived hits were preferred over other databases.

### Cryo-EM grid preparation, data collection, and image processing

Protein samples obtained from size exclusion were screened using negative-stain electron microscopy on a 120 kV JEM 1400 (JEOL). Samples were deposited on 400 mesh copper formvar/carbon film grids (Electron Microscopy Sciences) and stained with 2% uranyl acetate. Optimal fractions and concentrations were determined and used for cryo-EM grid preparation. R2/1 holey grids (Quantifoil) were coated with graphene oxide (Sigma–Aldrich). The sample (3 μl) was spotted and manually blotted for 4 s, followed by a plunge into liquid ethane using a CP3 plunger (Gatan). Datasets were collected on a 300 kV CryoARM microscope (JEOL) equipped with a K3 Summit direct electron detector (Gatan) at the VIB-VUB Bio-Electron Cryo-Microscopy Center. Movies were acquired at a magnification of 60 k in detector run in counting mode at a calibrated pixel size of 0.71 Å/pix, exposure of 60 e/Å for 61 frames. Prediction and fitting of molecular models in electron density maps was done using Phenix PredictandBuild (74). Three-dimensional reconstructions and molecular models were visualized using UCSF ChimeraX (75). Model to map correlation was performed using get_cc_mtz_pdb in Phenix (https://phenix-online.org/license) (43).

## Data availability

The cryo-EM electron density map has been deposited to the Electron Microscopy Data Bank and is available under the accession code: EMD-53314. The full dataset of bioinformatic and structural analysis is available through the GitHub account of the Iorga lab (https://github.com/IorgaLab/BSAP-Dataset) and the Zenodo open research repository (https://doi.org/10.5281/zenodo.15118892). The MS proteomics data have been deposited to the ProteomeXchange Consortium *via* the PRIDE partner repository with the dataset identifier PXD062775.

Requests for materials should be addressed to F.L.

## Supporting information

This article contains supporting information (65, 66, 76).

## Conflict of interest

The authors declare that they have no conflicts of interest with the contents of this article.
