# Supplementary material for: Identification of receptor-binding domains of Bacteroidales antibacterial pore-forming toxins
Source: J Biol Chem. 2025 Dec 29;302(2):111113. doi: 10.1016/j.jbc.2025.111113 (PMC12853789; doi:10.1016/j.jbc.2025.111113)
Supplement: Figures S1–S7 [file mmc1.pdf]

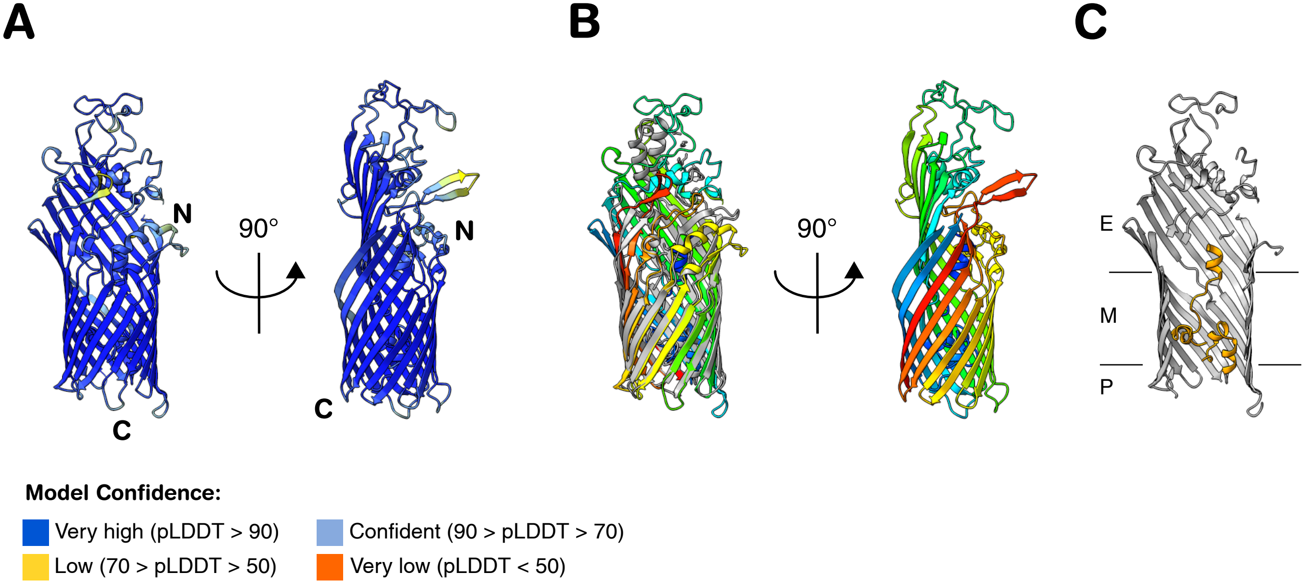

Figure S1. B1R^{S} is a structural homolog of the FadL family of proteins. *A*, AF3 model of the BSAP-1 receptor BF9343_1563 (GenBank: CAH07344.1). pLDDT is AlphaFold's per-residue confidence score, which scales from 0 to 100. *B*, Superimposition in cartoon representation of B1R^{S} (*chainbows colouring*) and *E. coli* FadL (*grey*, PDB 3dwn). *C*, The front of the β-barrel is cut away to show the N-terminal hatch domain (*orange*). P, periplasm; M, membrane; E, extracellular.

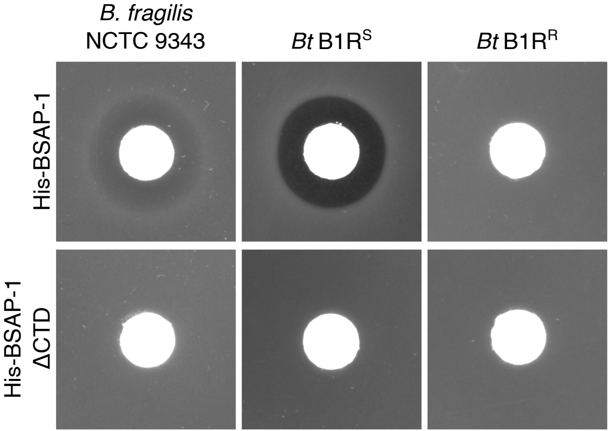

Figure S2. Activity assay of purified recombinant His-BSAP-1 and His-BSAP-1 ΔCTD. Growth inhibition of *B. fragilis* NCTC 9343, *B. thetaiotaomicron* B1R^{S} and *B. thetaiotaomicron* B1R^{R} strains grown in presence of 15 µg of purified His-BSAP-1 (*top row*) or His-BSAP-1 ΔCTD (*bottom row*). Representative results from at least three independent experiments are shown for each panel.

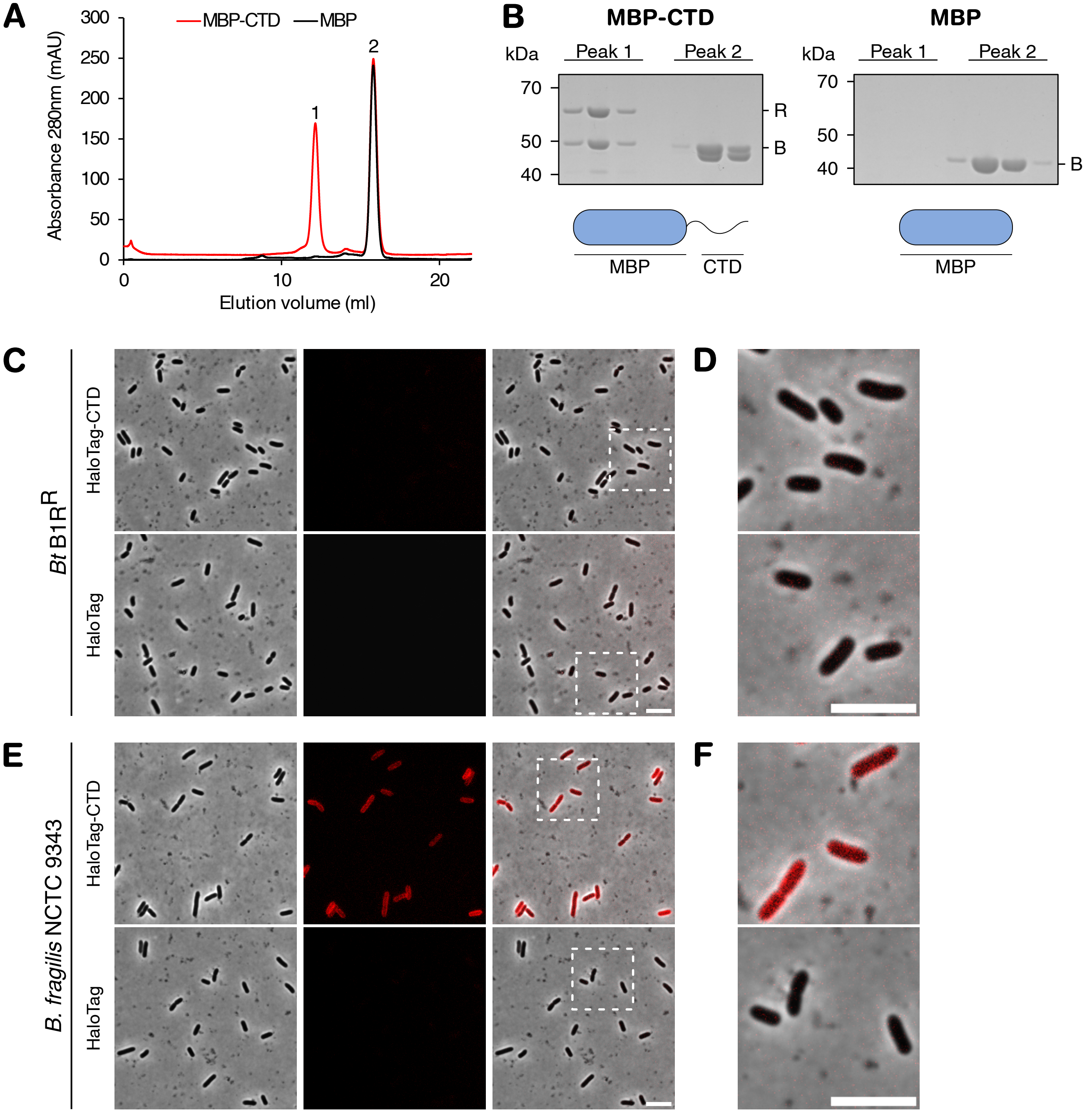

Figure S3. The BSAP-1 CTD is sufficient for receptor binding *in vitro* and *in vivo*. *A*, size-exclusion chromatography profile of affinity purified MBP-CTD (*red*) or MBP (*black*) following incubation with a B1R^{S}-containinig membrane fraction. *B*, Coomassie-stained gel showing the protein content of Peak 1 and Peak 2 from *A* for MBP-CTD (*left*) and MBP (*right*), respectively. Schematic representation of each protein is indicated below. MBP is in *blue*. B: bait protein; R: BSAP-1 receptor. *C*, HaloTag-CTD (*top*) or HaloTag (*bottom*) labelling of *B. thetaiotaomicron* B1R^{R} cells was visualized by epifluorescence microscopy. Scale bar represents 5 μm. *D*, zoom-in of cells boxed in *C*. Scale bar represents 5 μm. *E*, HaloTag-CTD (*top*) or HaloTag (*bottom*) labelling of *B. fragilis* NCTC 9343 cells was visualized by epifluorescence microscopy. Scale bar represents 5 μm. *F*, zoom-in of cells boxed in *E*. Scale bar represents 5 μm. Representative results from at least three independent experiments are shown.

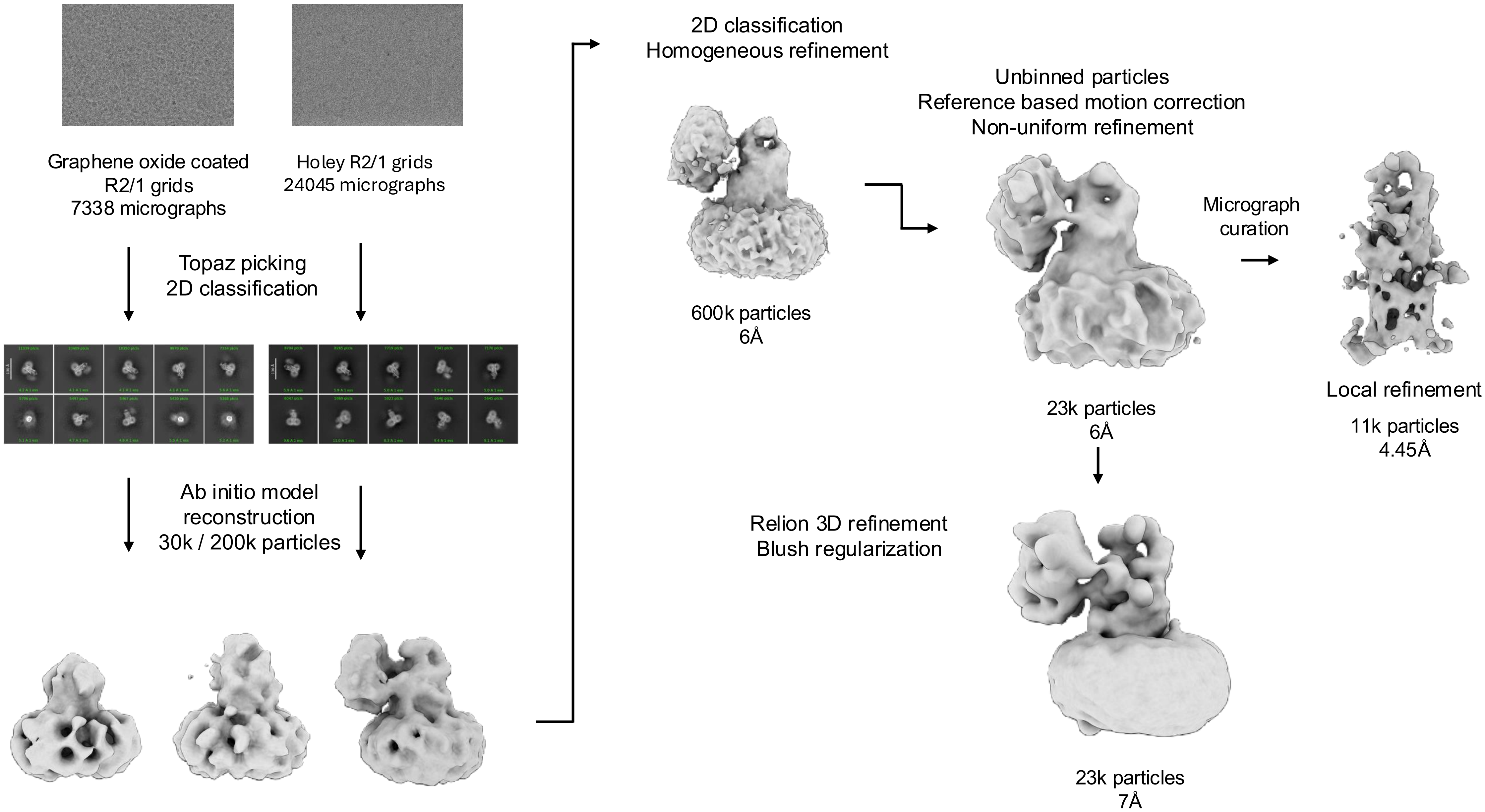

Figure S4. Cryo-EM Workflow for the BSAP-1 B1R^{S} complex structure. Image processing workflow.

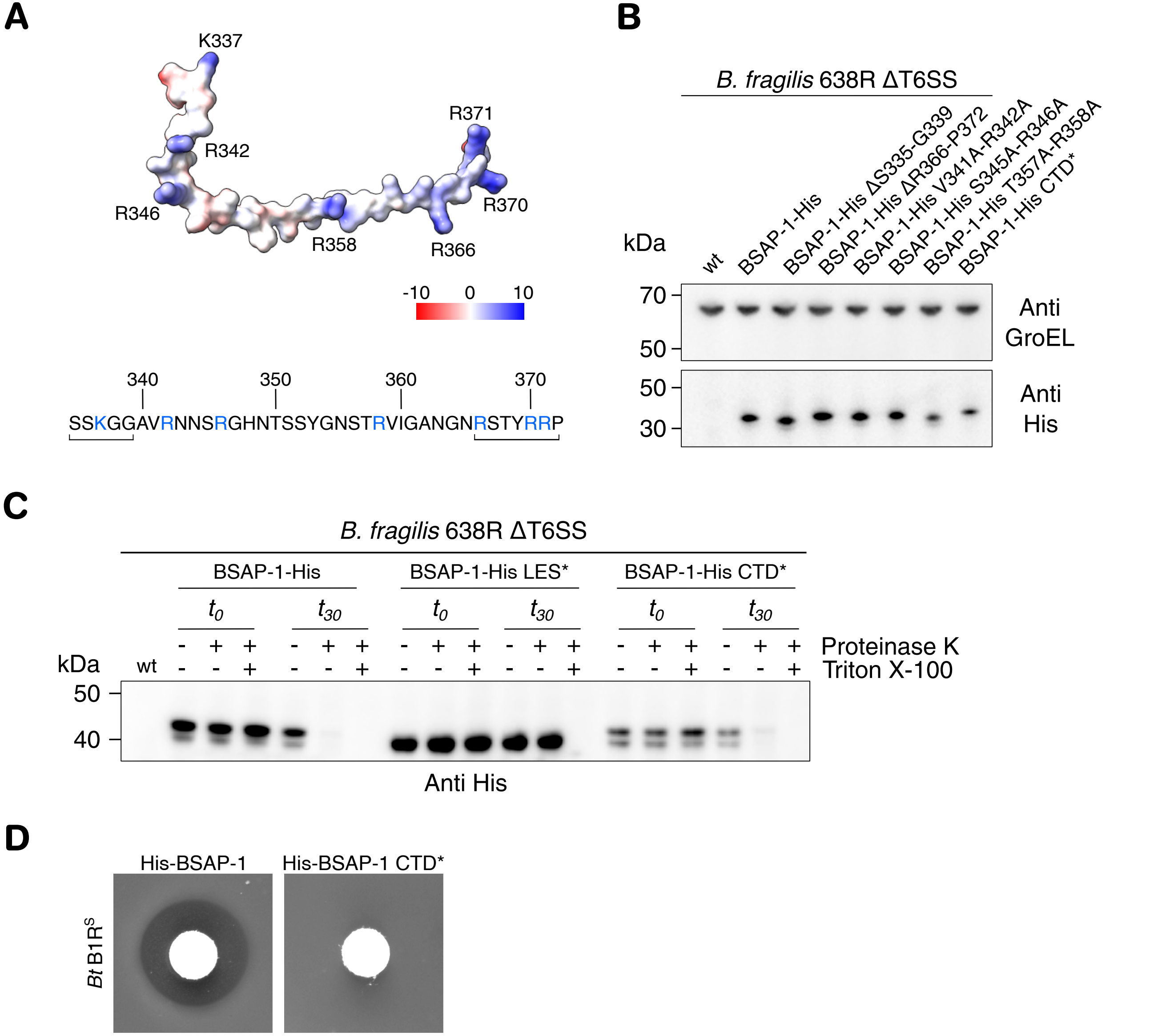

Figure S5. Positively charged CTD residues are critical for BSAP-1 bactericidal activity. *A*, Electrostatic potential (*top*) and amino acid sequence (*bottom*) of the BSAP-1 CTD; positively charged residues are indicated in *blue*, deletions are indicated *below*. *B*, whole-cell immunoblot analysis of the indicated *B. fragilis* 638R strains. Detection of GroEL was used as loading control. *C*, immunoblot analysis of the indicated Proteinase K treated *B. fragilis* 638R strains in absence or presence of the detergent Triton X-100. *D*, growth inhibition of *B. thetaiotaomicron* B1R^S grown in presence of 15 µg of purified His-BSAP-1 (*left*) or His-BSAP-1 CTD* (*right*). Representative results from at least three independent experiments are shown for each panel.

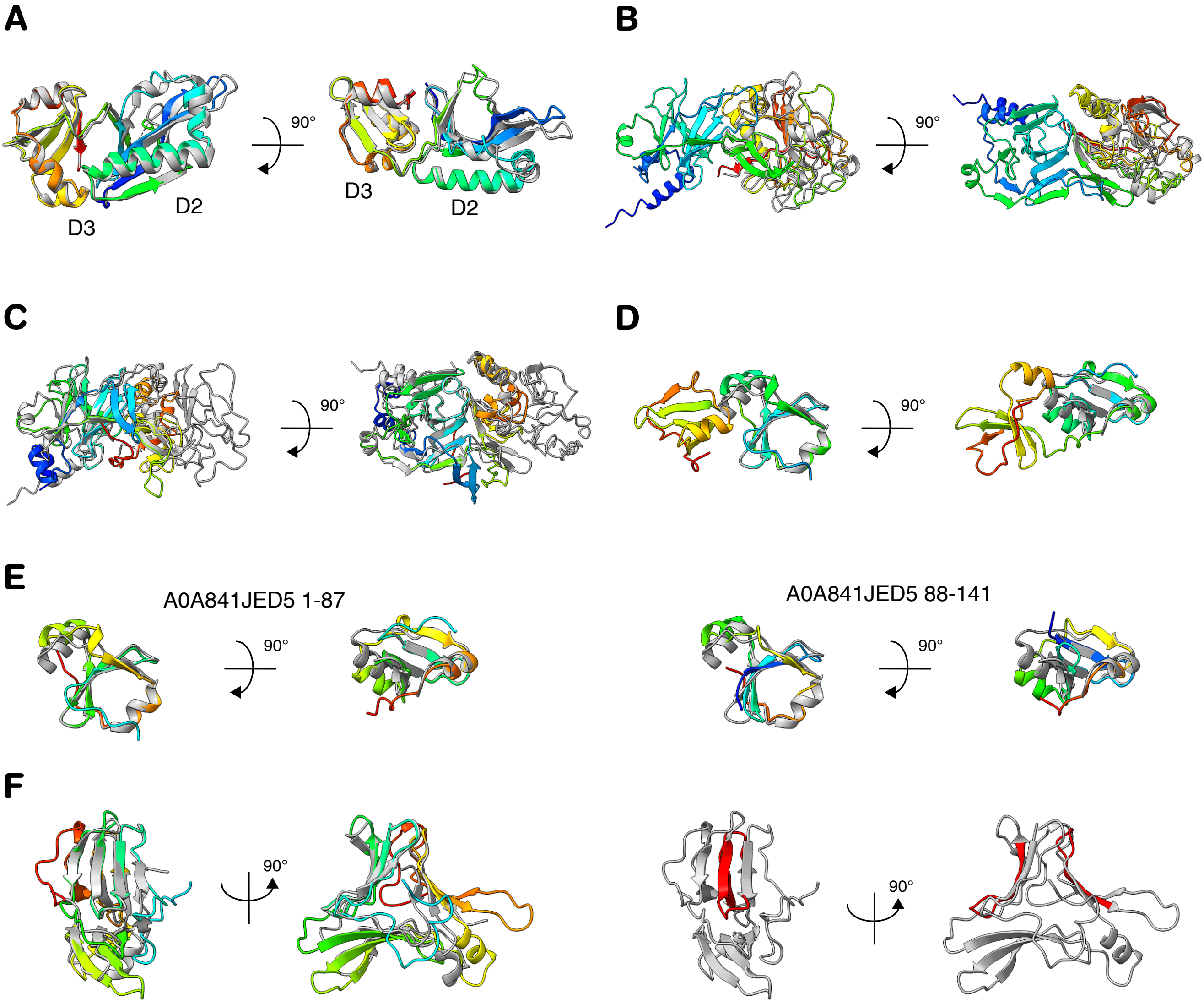

Figure S6. Structural features of Cluster 2 to 6 CTDs. *A*, Superimposition in cartoon representation of Cluster 2 representative A0A7T7HD40 (*chainbows colouring*) and Bt_3439 (*grey*, PDB 3kk7). Domains D2 and D3 are indicated. *B*, Superimposition in cartoon representation of Cluster 3 representative A0A1H0IJU1 (*chainbows colouring*) and the PF09603 representative A0A7C5V8K5 (*grey*). *C*, Superimposition in cartoon representation of Cluster 5 representative F3QYX7 (*chainbows colouring*) and Cluster 3 representative A0A1H0IJU1 (*grey*). *D*, Superimposition in cartoon representation of Cluster 4 representative A0A841JED5 full-length CTD (*chainbows colouring*) and the R1 repeat of $EccB_5$ (*grey*, PDB 7npr). *E*, Superimposition in cartoon representation of the first (*left*) and second (*right*) R1 repeat from Cluster 4 representative A0A841JED5 (*chainbows colouring*) and the R1 repeat of EccB5 (*grey*, PDB 7npr). *F*, Superimposition in cartoon representation of Cluster 6 representative A0A1H0PJ05 (*chainbows colouring*) and the D-rhamnose binding domain of pyocin L1 (*grey*, PDB: 4led) (*left*). The conserved QxDxNxVxY carbohydrate-binding motif is indicated in *red* (*right*).

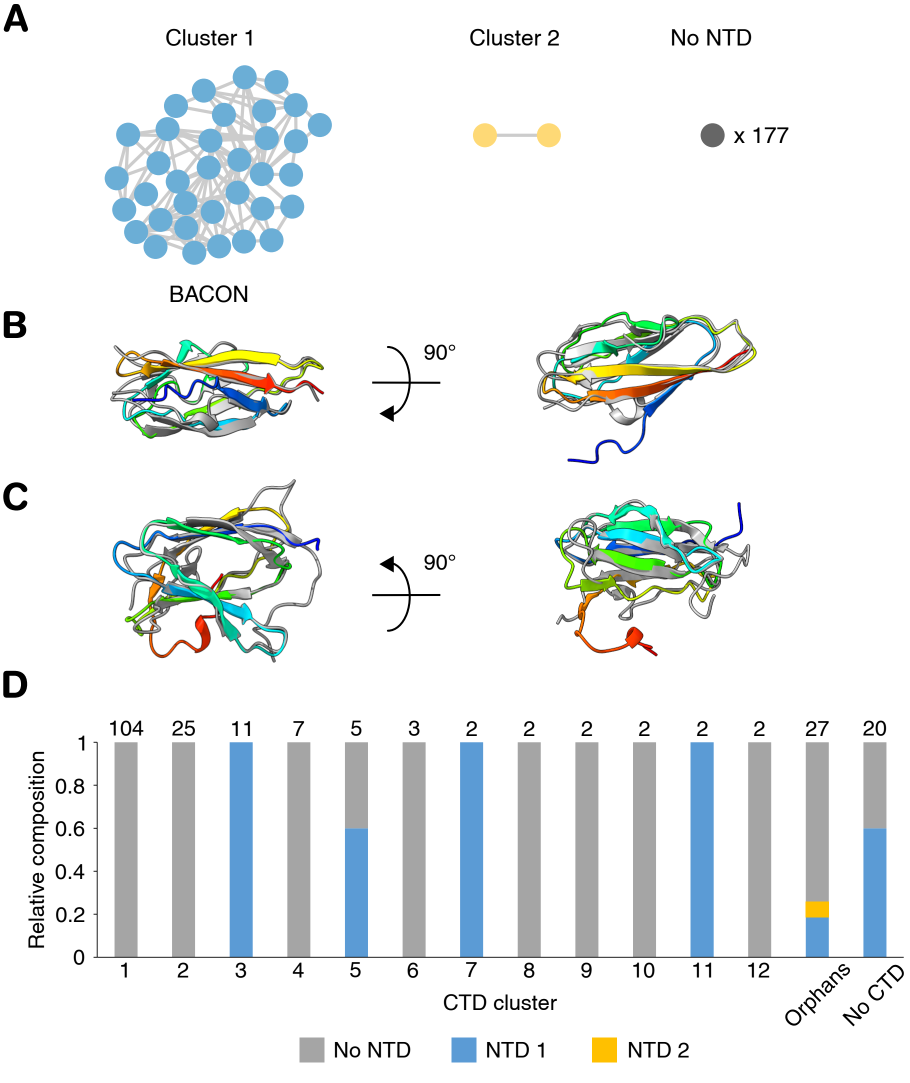

Figure S7. Clustering and structural features of Cluster 1 and 2 NTDs. *A*, network analysis of BSAPs clustered according to structural similarity of their NTDs. Where possible, Foldseek-derived CTD domain annotation is indicated below each cluster. *B*, side (*left*) and top (*right*) view of the structural comparison between the AlphaFold3 model of the NTD of E6SR77 (*chainbows colouring*) and the BACON domain of BoGH5A (*grey*, PDB: 3zmr). *C*, front (*left*) and side (*right*) view of the structural comparison between the AlphaFold3 model of the NTD of A0A9E3K7K4 (*chainbows colouring*) and the beta domain of pVHL (*grey*, PDB: 6hr2). *D*, bar chart representation of the co-occurrence of BSAP NTDs and CTDs. The number above each bar indicates the total number of representative sequences.
